# Mapping Rare Disease Registries in Brazil: Situational Analysis and Proposal for National Unification

**DOI:** 10.1007/s10916-026-02442-w

**Published:** 2026-07-20

**Authors:** Filipe Andrade Bernardi, Bibiana Mello de Oliveira, Natan Monsores de Sá, Domingos Alves, Têmis Maria Félix

**Affiliations:** 1 Faculty of Medicine, RISE-Health, MEDCIDS, University of Porto, Porto, Portugal; 2 Ribeirão Preto Medical School, University of São Paulo, Ribeirão Preto, Brazil; 3 Federal University of Health Sciences of Porto Alegre, Porto Alegre, Brazil; 4 Rare Disease Network (RARAS), Porto Alegre, Brazil; 5 Medical Genetics Service, Porto Alegre Clinical Hospital, Ramiro Barcelos St, Porto Alegre, RS 2350, 90610-910 Brazil; 6 Faculty of Health Sciences, University of Brasília, Brasília, Brazil

**Keywords:** Rare diseases, Health information systems, Public health policy, Health registries, Unified Health System, Epidemiology

## Abstract

Rare disease registries in Brazil remain fragmented across federal, state, and local initiatives, limiting the availability of reliable epidemiological information to support diagnosis, care planning, research, and public policy. This study aimed to map existing rare disease registry entities and registry-related initiatives in Brazil and to propose practical guidelines for their unification into an integrated national registry. We conducted a descriptive, exploratory mapping study combining a structured literature search with documentary analysis of public policies, health information systems, registry portals, institutional reports, and legislative documents related to rare diseases in Brazil. PRISMA-S was used to report the search component, and a PRISMA-style flow diagram documented source identification and selection. We identified a rapidly evolving legislative landscape, including federal bills proposing a national monitoring system or registry and recent state-level statutes related to identification and observatories. Using an expanded, auditability-oriented inventory definition, we mapped 28 registry entities and registry-related initiatives. Of these, 24 are implemented, three are legislative proposals, and one is under development. Among the 24 implemented initiatives, 16 are national or multicentre, Brazil-based initiatives; three are state-level; four are regional/local; and one is a transnational registry with documented participation of a Brazilian cohort. Registry creation and registry-related activity accelerated after 2018, particularly between 2020 and 2026. We conclude that Brazil exhibits substantial data fragmentation across uncoordinated systems. A unified approach should integrate epidemiological data from existing networks, state notification systems, specialised hospital registries, and technology appraisal information under coordinated governance, while embedding privacy-by-design and information security safeguards.

**Supplementary Information:**

The online version contains supplementary material available at 10.1007/s10916-026-02442-w.

## Introduction

Rare diseases (RDs) comprise a heterogeneous set of low-prevalence conditions with marked clinical diversity and high diagnostic complexity, posing a significant challenge to health systems worldwide [1]. In Brazil, the National Policy for Comprehensive Care for People with Rare Diseases (Política Nacional de Atenção Integral às Pessoas com Doenças Raras, PNAIPDR), established by Ordinance No. 199/2014, defines any condition affecting up to 65 individuals per 100,000 inhabitants as rare [2]. An estimated 6,000 to 8,000 RDs are known worldwide, of which approximately 80% are genetic in origin [3]. They affect about 6% of the population; in Brazil, this corresponds to roughly 13 million people living with rare diseases (PLWRD) [4, 5].

The rarity and complexity of RDs pose challenges, including delayed diagnosis, limited access to specific therapies, and the scarcity of robust epidemiological data to support evidence-based public policies [6]. Implementing structured RD registries is therefore essential for developing effective policies, ensuring appropriate resource allocation, and improving quality of care [7]. Nevertheless, the national scenario remains fragmented: registry initiatives coexist across different spheres (national, state, and regional) with diverse scopes and formats and insufficient standardisation, resulting in information gaps, duplication of efforts, and coordination difficulties [8].

In this context, the absence of a comprehensive, unified registry system is a significant barrier to advancing RD care in the country. The collection and organisation of information on prevalence and incidence, as well as on patients’ clinical, demographic, and geographic characteristics, are crucial to: (a) assess the magnitude of the problem; (b) plan and organise services; (c) optimise resource allocation; (d) foster research and the development of new therapies; and (e) ensure equitable access to medicines and treatments [9].

Alongside these epidemiological and information gaps, Brazil has progressively strengthened its health technology assessment and regulatory apparatus for RDs. Since its creation in 2011, the National Committee for Technology Incorporation (CONITEC) into the Sistema Único de Saúde (SUS, Brazilian Unified Health System) has appraised 790 technologies between 2012 and 2023, 164 of which were medicines for RDs. The most frequently assessed indications included multiple sclerosis (13.3%), cystic fibrosis (6.3%), and pulmonary hypertension (5.7%) [10]. In parallel, the Agência Nacional de Vigilância Sanitária (ANVISA, Brazilian Health Regulatory Agency) has implemented differentiated regulatory procedures for medicines intended for rare diseases. ANVISA reported that, in 2018, the average regulatory registration review time for rare-disease medicines was 170 days for biological products and 196 days for new synthetic medicines, compared with 520 and 515 days, respectively, for other medicines. These figures refer to the regulatory market-authorisation/registration process after submission/formalisation of the application, not to preclinical development or clinical trial phases [11, 12].

At the same time, the consolidation of national health registries must navigate an evolving data-protection and legal framework. Brazil’s General Data Protection Law (Lei Geral de Proteção de Dados Pessoais, LGPD) sets clear guidelines for processing sensitive health data, requiring appropriate legal bases, valid consent, or well-founded public-interest justifications for its use [13]. Any integration of RD registries must therefore incorporate privacy-by-design measures and robust information-security safeguards from the outset [13, 14]. For international readers, the LGPD is broadly comparable to the European Union’s General Data Protection Regulation (GDPR).

Current legislative initiatives already address these concerns: Bill No. 109/2025, which proposes a National System for Monitoring Rare Diseases, explicitly mentions aligning the registry system with the LGPD to ensure the confidentiality and ethical use of personal information [15]. Beyond the LGPD, a “new legal framework” for RDs is emerging.

In December 2023, the Health Committee of the Chamber of Deputies approved a substitute text for Bill No. 4058/2023 establishing the proposed Statute of the Person with a Rare Disease. As of 10 July 2026, the bill was ready for consideration by the Committee on Constitution, Justice and Citizenship (CCJC), following presentation of the rapporteur’s opinion on 7 May 2026. The substitute text would establish the National Registry of People with Rare Diseases (CadRaras), an electronic registry intended to identify people living with rare diseases, describe their socioeconomic profiles, and document barriers to the exercise of their rights. Under the proposal, CadRaras data would be used for public-policy formulation, evaluation, and research, subject to privacy safeguards [16].

Given this landscape, this study aims to map RD registry entities and registry-related initiatives currently identifiable in Brazil, analyse their characteristics, scope, and purposes, discuss the challenges arising from fragmentation and lack of standardisation, and propose guidelines for unification into an integrated national system. It is argued that such integration is imperative to strengthen epidemiological surveillance, inform public policies, and ultimately improve the quality of life of PLWRD in the country [7, 8].

## Methods

We conducted a descriptive, exploratory mapping study combining a structured literature search with documentary analysis of public policies, health information systems, registry portals, institutional reports, and legislative documents related to rare diseases in Brazil. PRISMA-S was used to transparently report the search component [17], and a PRISMA-style flow diagram was used to document source identification, Rayyan-assisted de-duplication, screening, eligibility assessment, and inclusion [18]. These reporting tools do not imply that the study was designed as a conventional systematic review of interventions, diagnostic accuracy, prognosis, or treatment effectiveness. The documentary component adhered to established procedures for qualitative document analysis [19].

Sources were identified through peer-reviewed and bibliographic databases (PubMed, Scientific Electronic Library Online (SciELO), Literatura Latino-Americana e do Caribe em Ciências da Saúde (LILACS), and Biblioteca Virtual em Saúde (BVS)) and grey literature issued by the Brazilian Ministry of Health, state health secretariats, legislative databases, registry portals, professional societies, patient organisations, and reference institutions. Searches covered 1 January 2000 to 14 June 2026 and used Boolean combinations in Portuguese, English, and Spanish, including (“doenças raras” OR “rare disease*”) AND (registro* OR registry OR “information system” OR “notificação”) AND (Brasil OR Brazil). Source-specific strategies, date limits, filters, duplicate-removal and screening fields, eligibility criteria, and reasons for exclusion are provided in Supplementary Table S2, in line with PRISMA-S reporting recommendations [17]. Database records were retrieved from PubMed (*n* = 288), SciELO (*n* = 88), LILACS (*n* = 80), and BVS (*n* = 83), totalling 539 records. The 539 records refer exclusively to the four bibliographic databases. In parallel, 22 included URL-level source bundles linked to the mapped registry entities were identified through official, legislative, governmental, registry, professional-society, patient-organisation, and institutional websites. Records were exported and imported into Rayyan for pooled duplicate detection and screening management. Duplicate removal was performed globally after pooled import; therefore, duplicate counts were not apportioned by database/source.

Eligibility criteria encompassed registries, information systems, databases, administrative case lists, legislative proposals, programme-generated datasets, specialty-led registries, mixed-scope databases, umbrella registry platforms, or transnational registries with documented Brazilian participation when they had a defined rare-disease or rare-disease-relevant scope; were national, state-level, regional/local in Brazil, or included Brazilian participation; were implemented, proposed, or under development between 1 January 2000 and June 2026; and were corroborated by an official document, institutional source, or scientific publication. Two reviewers independently screened titles/abstracts or document titles and then full texts or source documents, resolving discrepancies by consensus; duplicates were removed before eligibility assessment. A PRISMA-style flow diagram summarises identification, screening, eligibility assessment, and inclusion [18]. Supplementary Figure S1 summarises the database/source identification and selection flow, including Rayyan-assisted duplicate removal, screening, eligibility assessment, reasons for exclusion, and final inclusion. Dataset S2 provides the included-source auditability log for the 28 included sources.

Data extraction used a standardised spreadsheet capturing: system name, year of creation, territorial coverage, primary purpose, implementation status, and operational characteristics. Where available, we also recorded governance/responsible body, data sources, case definition, core data elements, update frequency, and interoperability standards. We triangulated information across multiple sources and, when feasible, contacted custodians to clarify ambiguities. The complete auditable inventory is provided in Supplementary Table S1, and the machine-readable mapping spreadsheet is provided as Dataset S1.

Descriptive statistics (counts, proportions) and a temporal analysis of registry creation were performed to characterise trends. Ethics approval was not required because only public-domain data and official documentary sources were analysed. Procedures for document analysis and source appraisal followed recognised methodological guidance [19].

To facilitate analysis, initiatives were classified into three status categories: implemented registries or registry-related initiatives (operational systems, platforms, databases, lists, or programmes that routinely capture or generate case-based data), proposals (legislative or institutional initiatives not yet operational), and initiatives under development (projects with defined scope and custodianship that have not yet started routine data collection). Territorial scope was coded as national/multicentre, state-level, regional/local, or international/Brazil cohort according to the initiative’s primary jurisdiction and documented Brazilian participation. The unit of analysis was an “RD registry entity or registry-related initiative,” which could include population-based programmes (e.g., the national newborn screening programme), mixed-scope databases, specialty-led registries, and umbrella platforms that produce case-level datasets on rare or rare-disease-relevant conditions. Umbrella platforms and mixed-scope databases were counted once at the platform or database level, rather than split into individual embedded projects, to avoid denominator inflation. Transnational registries were counted only when Brazilian participation or a Brazilian cohort was explicitly documented and were reported separately by scope. General electronic medical records or generic event-notification systems were excluded unless they contained a documented RD-specific module, case-identification component, or data flow.

## Results

Bibliographic database searches identified 539 records. After removal of 140 duplicates, 399 database records were screened; 335 were excluded and 64 database-derived full texts were assessed. Of these, 58 were excluded and six database-derived extraction units were included. In parallel, 22 URL-level source bundles linked to the mapped registry entities were screened, assessed for eligibility, and included. Overall, 561 records/source bundles were identified, 421 were screened, 86 were assessed for eligibility, and 28 extraction units were included, comprising six database-derived units and 22 URL-level source bundles.

### Legislative Landscape

During the review period, three federal bills were identified that aim to establish a national registry or monitoring system for RD within Brazil’s Unified Health System (SUS). Bill No. 109/2025 proposes the National System for Monitoring Rare Diseases (Sistema Nacional de Monitoramento de Doenças Raras – SNMDR), which specifies mandatory notification events and requires that data processing comply with the General Data Protection Law (Lei Geral de Proteção de Dados Pessoais – LGPD). Bill No. 3373/2025 creates a National Registry of Rare Diseases, sets a timeframe for a technical normative definition of “rare disease”, and regulates access and consultation. Bill No. 4197/2025 likewise establishes the SNMDR, emphasising the need for a national database integrated with research institutions and reference centres [13, 15, 20, 21].

Despite differences in terminology (“SNMDR” versus “National Registry of Rare Diseases”) and institutional arrangements, the three bills converge on core pillars: (i) standardised compulsory notification (including congenital anomalies at birth, confirmed positive neonatal screening results, and diagnoses at any level of care); (ii) conditionality of access to benefits (e.g., high-cost medicines and selected public programmes) on prior registry enrolment; and (iii) explicit data-protection safeguards aligned with the LGPD [13, 15, 20, 21]. According to Chamber of Deputies records, rechecked at resubmission, Bill No. 109/2025 remained pending the rapporteur’s opinion in the Committee on Constitution, Justice and Citizenship (CCJC) after approval by the Health Committee. Bill No. 3373/2025 was being processed under urgent procedure and was ready for the Plenary agenda, with a Plenary rapporteur designated, while also listed as awaiting rapporteur designation in the Health, Finance and Taxation, and Constitution and Justice committees. Bill No. 4197/2025 remained awaiting the rapporteur’s opinion in the Health Committee [15, 20, 21].

In parallel with these federal initiatives, some states have begun developing their own legal frameworks for people living with RD. A prominent example is the State of Rio de Janeiro, which in 2024 enacted Law No. 10.315/2024, establishing the State Statute of the Person with a Rare Disease. This statute consolidates rights, mandates the dissemination of information on available benefits and services, and includes measures of financial support for families, signalling how subnational legislation can anticipate and complement national efforts [22].

Taken together, the three federal bills constitute a timely legislative window to consolidate dispersed RD initiatives into a single national registry within SUS. A nationally unified registry should be designed to align with this emerging framework by embedding standardised, compulsory reporting, federated governance, and robust data protection requirements. It should also prevent normative overlap among parallel initiatives by integrating existing state and specialised databases under a single interoperability model, with clear rules for access, security, and auditability [13, 14, 15, 20, 21].

### Overview of the Identified Registry Entities and Registry-Related Initiatives

The 28 included extraction units supported the identification of 28 RD registry entities and registry-related initiatives involving Brazil (Supplementary Table S1; Dataset S1; Dataset S2). Of these, 24 (85.7%) are implemented, three (10.7%) are legislative proposals, and one (3.6%) is under development. Among the 24 implemented initiatives, 16 are national or multicentre Brazil-based initiatives, three are state-level, four are regional/local, and one is a transnational registry with documented Brazilian cohort participation (66.7%, 12.5%, 16.7%, and 4.2% of implemented initiatives, respectively). Within the total, four national-level entries remain at the proposal or development stage: three federal bills and one technical initiative. A temporal analysis indicates an inflection after 2018, with acceleration between 2020 and 2026, although the expanded denominator includes older specialty-led and condition-specific registries that were previously described narratively.

#### National Initiatives

Established in 2020, the Brazilian Rare Diseases Network (RARAS Network) is a multicentre project involving 40 institutions across Brazil’s five regions. In its national epidemiological survey (*n* = 12,530), it reported a median age of 15 years and a predominance of phenylketonuria, cystic fibrosis, and acromegaly, with a mean “diagnostic odyssey” of 5.4 years [5]. In parallel, the platform reports 62 participating researchers, 19,458 registry records, and 2,391 RDs, indicating a continuous effort to compile and analyse national data [3].

As a result of this evolving landscape of RD research in Brazil, the National Institute for Rare Diseases (Instituto Nacional de Doenças Raras – InRaras) was created in 2023 as a National Institute of Science and Technology (Instituto Nacional de Ciência e Tecnologia, INCT). Its objectives include developing strategies and mechanisms to accelerate diagnosis, evaluating approaches to optimise and reduce diagnostic costs, and promoting training and scientific dissemination activities [23]. Both initiatives (RARAS and InRaras) are headquartered at the Hospital de Clínicas de Porto Alegre (HCPA). Together, these initiatives also create a national infrastructure for genomic and biochemical investigation of suspected RDs, which can later be integrated with registry data [24].

Launched in August 2024, the Raras Brasil Project constitutes the first self-administered national registry of PLWRD, resulting from a collaboration between the University of Brasília (UnB), the Ministry of Human Rights and Citizenship, and the Ministry of Health. The Observatory of Rare Diseases collects biopsychosocial information through self-administered questionnaires to produce a situational diagnosis to inform public policies and social support strategies [4].

In addition to these RD-specific initiatives, Brazil has long-standing population-based information systems that effectively function as extensive registries for selected congenital and rare conditions. The congenital anomaly surveillance system is built primarily on the Live Birth Information System (Sistema de Informações sobre Nascidos Vivos – SINASC) and the Mortality Information System (Sistema de Informações sobre Mortalidade – SIM). Since 2010, SINASC has systematically recorded congenital anomalies in live births and currently captures approximately 2.9–3.0 million births per year, with national coverage approaching 98% [25]. Analyses of national and international registries indicate that, owing to this high coverage and volume, the Brazilian congenital anomaly surveillance system based on SINASC is among the largest population-based congenital anomaly surveillance systems worldwide [25, 26].

The National Neonatal Screening Programme (Programa Nacional de Triagem Neonatal, PNTN) also plays a central role in early identification and longitudinal follow-up of several rare conditions [27]. Established in 2001 as a population-based “heel prick test” programme within the SUS, the PNTN initially screened for phenylketonuria and congenital hypothyroidism, and was later expanded to include sickle cell disease and other haemoglobinopathies, cystic fibrosis, congenital adrenal hyperplasia, and biotinidase deficiency [28]. Recent national analyses show public coverage of around 80–95% of live births, with some states approaching universal screening [29]. Beyond its clinical impact, the PNTN generates structured data on newborns diagnosed through screening, serving as a national registry for several rare metabolic, endocrine, and haematological diseases.

Another technological front is the integration of genomic data from PLWRD. The Genomas Brasil Programme, launched in 2020, aims to sequence 100,000 genomes from Brazilian individuals, including patients with RD, and to build a national genetic data repository [30]. With a planned investment of approximately BRL 600 million, the initiative seeks to link genetic variants to disease phenotypes and support the development of personalised therapies for rare conditions. In 2025, the Ministry of Health, in partnership with Fiocruz Bahia, proposed a national genomic data repository and platform to integrate existing databases and genetic cohorts nationwide. This effort defines governance, technical requirements, and processes for a unified genomic information infrastructure, ensuring data security while enabling research and innovation in precision health [31].

In addition to these broad national initiatives, the expanded inventory includes a heterogeneous set of condition-specific, specialty-led, mixed-scope, umbrella, and transnational registry-related initiatives, many of them coordinated by professional societies, patient organisations, or academic networks (Table 1). These initiatives were counted when they had a documented registry, database, or case-based data-collection function and a rare-disease or rare-disease-relevant scope. Mixed-scope and umbrella platforms were counted once at the platform or database level to avoid inflating the denominator by splitting embedded subprojects.

**Table 1:** Condition-specific, specialty-led, mixed-scope, umbrella, and transnational registry-related initiatives included in the expanded inventory

Registry/initiative	Main condition(s) or scope	Coordinator/type
Brazilian Cystic Fibrosis Registry (REBRAFC)	Cystic fibrosis	Brazilian Cystic Fibrosis Study Group (GBEFC) – professional society [32]
National Spinal Muscular Atrophy Registry (Cadastro Nacional de AME)	Spinal muscular atrophy	National Institute of Spinal Muscular Atrophy (INAME) – patient organisation [33]
COMDORA-SBN rare kidney disease registries	Fabry disease; atypical haemolytic uraemic syndrome; cystinosis	Rare Diseases Committee of the Brazilian Society of Nephrology (COMDORA-SBN) – grouped specialty-led rare kidney disease registry entity [34]
Brazilian Sjögren’s Syndrome Registry (BRAS/REBRASS)	Primary Sjögren’s syndrome	Brazilian Society of Rheumatology – professional society [35]
BiobadaBrasil	Rheumatic diseases treated with biologic/targeted therapies, including rare-disease-relevant conditions	Brazilian Society of Rheumatology – professional society [36]
Brazilian Prospective Hodgkin Lymphoma Registry	Hodgkin lymphoma	Academic haematology centres, including Federal University of Rio de Janeiro – multicentre cohort [37]
National Amyotrophic Lateral Sclerosis Registry	Amyotrophic lateral sclerosis (ALS)	revELA project (LAIS/UFRN), Ministry of Health, and ABrELA – academic–patient partnership [38]
REDONE.br – Brazilian Registry of Neurological Diseases	Neurological diseases, including rare neurological conditions	Brazilian Academy of Neurology (ABN) – professional society [39]
Cranio-Face Brazil / Brazilian Database on Orofacial Clefts	Orofacial clefts and craniofacial anomalies, including rare/syndromic conditions	Multicentre academic network (Cranio-Face Brazil Project) [40]
National Epidermolysis Bullosa Registry	Epidermolysis bullosa	DEBRA Brasil – patient organisation [41]
National Patient Registry – Brazilian Organisation for Crohn’s Disease and Colitis	Inflammatory bowel disease (Crohn’s disease, ulcerative colitis)	GEDIIB – professional society [27]
LASID Registry – Brazilian cohort	Primary immunodeficiencies	Latin American Society for Immunodeficiencies and participating Brazilian centres [42]

Note: Table 1 includes entries counted in the expanded inventory under the inclusive registry-related initiative rule. Umbrella or mixed-scope initiatives are counted once at platform/database level and labelled accordingly in Supplementary Table S1 and Dataset S1

#### Condition-Specific and Specialty-Led Registries

The National Spinal Muscular Atrophy Registry, coordinated by the National Institute of Spinal Muscular Atrophy (Instituto Nacional da Atrofia Muscular Espinhal, INAME), aggregates clinical and therapeutic information on patients with spinal muscular atrophy to inform public policies, access to specialised care, and research agendas [33]. Several specialty-specific initiatives focus on rare renal, rheumatological, and autoimmune diseases. The Rare Diseases Committee of the Brazilian Society of Nephrology (Comitê de Doenças Raras da Sociedade Brasileira de Nefrologia, COMDORA-SBN), which coordinates national registries for rare kidney diseases, also leads registries for Fabry disease, atypical haemolytic uraemic syndrome, and cystinosis, which support Brazilian consensus recommendations for diagnosis and management and contribute to improvements in clinical practice [34].

Within rheumatology, the Brazilian Sjögren’s Syndrome Registry (Registro Brasileiro de Doença de Sjögren – BRAS/REBRASS), coordinated by the Brazilian Society of Rheumatology, provides detailed clinical and epidemiological data on patients with primary Sjögren’s syndrome and has generated national analyses on disease profile and complications [35]. The Brazilian Registry for Monitoring Biologic Therapies in Rheumatic Diseases (BiobadaBrasil), also promoted by the Brazilian Society of Rheumatology, systematically follows patients receiving biologic and targeted therapies, producing evidence on their safety and effectiveness in routine care [36].

Oncology and neurology likewise contribute essential RD-related registries. The Brazilian Prospective Hodgkin Lymphoma Registry, also reported as the Brazilian Prospective Registry, is a national specialty-led web-based cohort/registry that collects clinical, treatment, and outcome data on Hodgkin lymphoma and has generated peer-reviewed outputs [37]. The National Amyotrophic Lateral Sclerosis Registry (Registro Nacional de Esclerose Lateral Amiotrófica), developed in the context of the revELA project in partnership with the Ministry of Health and the Brazilian Association of Amyotrophic Lateral Sclerosis (ABrELA), aims to build a population-based dataset of individuals living with ALS in Brazil [38]. The Brazilian Registry of Neurological Diseases (REDONE.br), an initiative of the Brazilian Academy of Neurology (ABN), is a national platform designed to describe the epidemiological profile of neurological diseases through continuous, standardised data collection [39].

Other initiatives illustrate how academic networks and patient organisations help fill information gaps for specific rare conditions. The Cranio-Face Brazil Project, a multicentre collaboration active since 2003, developed and maintains the Brazilian Database on Orofacial Clefts, which supports research and service planning for craniofacial anomalies [40]. The National Epidermolysis Bullosa Registry, coordinated by DEBRA Brasil (Dystrophic Epidermolysis Bullosa Research Association – DEBRA Brasil), the national association for people living with epidermolysis bullosa, has collected online data on more than 900 individuals with epidermolysis bullosa since 2014, with the explicit aim of estimating the number and geographic distribution of cases and supporting public policies and clinical trials [41]. The National Patient Registry of the Brazilian Organisation for Crohn’s Disease and Colitis (GEDIIB) consolidates data on inflammatory bowel disease and was retained under the expanded inclusive inventory as a mixed/specialty-led patient registry-related initiative illustrating how professional-society registries contribute to Brazil’s broader real-world data and registry-governance ecosystem [27].

Together, these registries reveal a vibrant but fragmented landscape. Most are disease-specific, use heterogeneous inclusion criteria and data models, and are not formally integrated with SUS information systems or with broader rare-disease policies. In addition, numerous patient associations (for example, those dedicated to osteogenesis imperfecta, fragile X syndrome, and other conditions) maintain small registries or membership databases that further expand available information but remain largely invisible to national planning processes. Systematically mapping and harmonising these initiatives is therefore a crucial prerequisite for building a unified national rare-disease registry architecture.

In addition to national initiatives, Brazilian patients also contribute to international disease- specific registries. An important example is the registry of the Latin American Society for Immunodeficiencies (LASID), which aggregates standardised data on primary immunodeficiencies across Latin America. Brazil is among the largest national cohorts within the LASID Registry, with more than 2,000 registered participants, reflecting the country’s active engagement in regional collaborative surveillance and research on rare immunological disorders [42].

#### Subnational Registries and Emerging Local Initiatives

At the subnational level, we identified one fully implemented state system in Paraná: the Sistema web para Notificação de Síndromes e Doenças Raras do Paraná (SIDORA). This online notification platform issues a personal card with a QR code and underpins the state-level RD registry infrastructure. Implemented in 2020, SIDORA serves as a digital notification and case-management system for PLWRD. Health services enter nominal data into the platform, and the system issues a personalised identification card with a QR code that can be presented at different points of care to signal the person’s condition and specific needs. Under State Law No. 21.240/2022, notification of RDs to SIDORA is compulsory, and the State Health Department (Secretaria de Estado da Saúde, SESA) must validate each record within ten working days [43]. Thus, the identification card is not merely symbolic; it is generated from and, in turn, strengthens a state-level registry infrastructure.

Other states have adopted instruments that partially overlap with registry functions but do not yet constitute comprehensive, interoperable systems. In São Paulo, State Law No. 17.802/2023 created an identification card for PLWRD, designed to guarantee preferential access and facilitate recognition of their rights within public services [44]. In Minas Gerais, State Law No. 25.351/2025 formally recognised a specific identification lanyard (“cordão de identificação”) for people with RDs, a pioneering initiative in the country [45]. Both measures rely on local administrative lists of eligible individuals, but available documents do not describe a structured state registry with standardised variables, regular updates, or integration with national systems.

Beyond these legal instruments, selected regional reference services and care pathways were retained conservatively as registry-related initiatives when public documentation or author-confirmed institutional information supported an identifiable service-embedded database, administrative list, case-tracking component, or structured data flow relevant to rare-disease care. Casa dos Raros was retained as a service-embedded rare-disease database/case-tracking component because peer-reviewed documentation describes a structured rare-disease service model, patient navigation, registry-related activities, and preliminary operational results. The APAE network entry was retained conservatively as a reference-service case-tracking initiative because public documentation supports rare-disease reference-service workflows, while structured database or case-list functionality was classified on the basis of author-confirmed institutional information rather than a publicly auditable formal registry document. In Santa Catarina, the epidermolysis bullosa care pathway was retained as a care-pathway-generated data-flow initiative because official documentation defines registration, evaluation forms, eligibility, referral, authorisation, and follow-up flows for people with epidermolysis bullosa. These entries are not interpreted as formal state-wide registries comparable to SIDORA [43, 46, 47].

Finally, we identified emerging data-driven initiatives that, although not registries in a strict sense, illustrate how subnational systems are beginning to use existing health information to generate structured lists of RD candidates. In the Federal District, for instance, the TAMIS-IA platform was developed as an intelligent triage tool that integrates data from primary care and hospital electronic health records and applies machine learning algorithms (Random Forest, XGBoost, SVM) to flag patients with a high probability of selected rare and chronic conditions [48]. The resulting lists can be reviewed by clinical teams and, in principle, could be used as a feeder mechanism for future registry structures.

Overall, outside Paraná, subnational initiatives remain focused primarily on service organisation and the recognition of rights, with only incipient or fragmented registry components. This contributes to the persistence of data fragmentation at state and regional levels and limits the potential for these experiences to feed a unified national RD registry architecture consistently.

### Integration with International Platforms

The global nature of RDs makes it essential that Brazilian efforts be integrated with consolidated international networks and platforms. A central reference is Orphanet, the European portal for RDs, which provides a comprehensive disease database, classifications (ORPHA codes), and a list of worldwide registries [1, 49, 50]. The adoption of internationally standardised classifications, such as the Orphanet nomenclature or the 11th revision of the International Classification of Diseases (ICD-11) of the World Health Organization (WHO), which incorporates numerous RDs, is strategic to ensure that Brazilian data can be compared and interoperable with data from other countries [51]. For example, the National Rare Disease Database (BNDMR) uses ORPHA codes to identify conditions, ensuring data comparability and facilitating integration with external databases [52]. Brazil can benefit from aligning its registries with these standards, enabling national information to be fed into global repositories and, in turn, to receive inputs from them.

Engagement with initiatives led by the WHO and the United Nations is also underway. In 2025, the Brazilian government followed a recommendation from the Federal Senate and formally supported a World Health Assembly resolution on “Rare diseases: a global priority for equity and inclusion in health” [53, 54]. This role as a co-sponsor of the WHO resolution signals a commitment to international collaboration, whether by sharing anonymised data for global RD surveillance or by adhering to best-practice guidelines developed jointly with other countries.

Global collaboration can also help Brazil address challenges such as access to orphan medicines, evaluation of high-cost technologies, and participation in multinational clinical trials. Experiences from Canada and Australia show that national strategies for RDs increasingly rely on coordinated registry infrastructures and data-sharing arrangements across regions and disease groups [55, 56, 57, 58].

In the European Union (EU), integrated strategies provide a helpful model. Since 2017, EU Member States have maintained European Reference Networks (ERNs) that connect centres of expertise for RDs, coupled with tools such as the Clinical Patient Management System (CPMS) for teleconsultation. These developments converge in the European Rare Disease Registry Infrastructure (EU RD Platform), which provides a set of common data elements and tools to harmonise data collection and support the creation of interoperable registries [59]. European data platforms such as the EU RD Platform and Orphadata provide resources to standardise data models, apply ORPHAcodes, and support registry governance [50, 59].

Brazilian initiatives can benefit from these experiences by progressively aligning national and local registries with international standards and promoting interoperability with global platforms. In this context, an emerging Lusophone initiative is mapping RD registries and data infrastructures across Portuguese-speaking countries to support shared policies and joint research efforts among Brazil, Portugal, and African Portuguese-speaking countries, reinforcing the need for interoperable architectures and common minimum data sets [60, 61].

## Discussion

### Fragmentation and Gaps in Brazilian RD Registries

The mapping conducted in this study reveals considerable fragmentation of RD registry entities and registry-related initiatives in Brazil, characterised by the coexistence of multiple systems with specific purposes and limited integration. This pattern is consistent with international findings indicating that countries with federal arrangements tend to exhibit dispersed information structures and coordination challenges [62]. A temporal concentration in the creation of registry entities and registry-related initiatives was also observed from 2018 onwards, with a notable acceleration between 2020 and 2025, suggesting institutional maturation and increased prioritisation of the topic on the public agenda, partly catalysed by the PNAIPDR and growing awareness of the epidemiological and social relevance of these conditions [2, 8].

At the national level, the RARAS Network represents a significant advance by producing pioneering epidemiological data at scale, including an estimate of a mean “diagnostic odyssey” of 5.4 years [5]. International reports, predominantly from high-income settings such as the United States and Europe, often describe diagnostic intervals of around 6–8 years or longer [62, 63]. Comparisons between these global estimates and the RARAS-BR figure should therefore be interpreted with caution, as differences may reflect methodological heterogeneity, distinct sample profiles, or recent improvements in Brazilian care pathways. Regardless of the exact magnitude, the gap between the need for and availability of specialised diagnostic services remains substantial and underscores the urgency of professional training, the standardisation of care pathways, and the strengthening of reference centres. The absence of a structured, compulsory national system for the registration of RDs, as identified in this study, corroborates the literature highlighting the insufficiency and fragmentation of existing databases that support the planning and evaluation of public policies [5].

From a regulatory and governance perspective, Bill No. 109/2025 proposes establishing the SNMDR within the SUS, in compliance with the LGPD. It makes registry enrolment a condition for access to high-cost medicines, participation in social programmes, and inclusion in clinical research [15]. In a fragmented context, the absence of a unified, accessible database may create additional bureaucratic barriers to proving eligibility, thereby exacerbating vulnerabilities and delaying the start of potentially disease-modifying treatments. The scientific dimension is equally affected: research on RDs depends on large, well-characterised cohorts to understand natural history, validate biomarkers, and test interventions; the dispersion of registries complicates recruitment, analytical reproducibility, and Brazil’s participation in international multicentre networks [12].

The expected gains from a unified architecture are multiple and interdependent. From a care perspective, information consolidation tends to shorten the diagnostic odyssey, orient care pathways, and improve longitudinal coordination, promoting more timely access to therapies and rehabilitation. For research, a standardised, access-controlled and pseudonymised repository increases the critical mass of data, accelerates the identification of eligible cohorts, and supports Brazil’s participation in international consortia, potentially shortening the cycle of technology development and incorporation. In public management, the availability of robust, comparable indicators improves policy planning, monitoring, and evaluation, thereby enhancing resource allocation and mitigating regional asymmetries. From an equity perspective, a national registry facilitates the verification of eligibility for high-cost medicines and social benefits while simultaneously strengthening patient and advocacy organisations by consolidating evidence.

### Expected Impacts and Practical Applications

In clinical research, a unified registry significantly enhances the ability to identify study and therapeutic trial candidates. Cases that previously remained “invisible” within the health system can, through the registry, be located and invited to participate in research on new medicines or advanced therapies. This is particularly relevant in RDs, where recruiting sufficient numbers of patients is a significant barrier to treatment development. With a robust national database, Brazil also becomes a more attractive partner in international research consortia, able to contribute data to multicentre studies and, in return, benefit from therapeutic innovations (e.g., expanded access to experimental medicines through international protocols).

Regarding access to advanced therapies, the national registry can support more efficient policies for coverage and financing. By accurately quantifying the number of patients with each RD and their geographic distribution, policymakers can better estimate potential demand for high-cost medicines (such as recombinant enzymes, gene therapies, or molecularly targeted drugs) and negotiate improved pricing and supply conditions with pharmaceutical companies. The recent launch of Canada’s National Strategy for Drugs for Rare Diseases, for example, emphasises data collection and the use of evidence for coverage decisions, including the development of standards to improve RD registry data [55, 56]. Similarly, in Brazil, consolidated data could inform updates to the SUS exceptional medicines list and resource allocation from the National Health Fund, ensuring that innovative therapies reach those in need more equitably.

In territorial planning of services, epidemiological maps derived from the registry will help identify regions with higher concentrations of specific RD groups or service gaps. This supports the rational expansion of reference centres nationwide, avoiding unnecessary service duplication in some areas while extending specialised care to underserved regions. For instance, if data indicate a high number of lysosomal disease cases in the North/Northeast, establishing a reference centre in those regions becomes justified, reducing the need for long-distance travel by patients. Moreover, standardised information on the care pathway (patient journey) allows assessment of the effectiveness of existing care lines. It supports proposals to improve integration of primary, specialised, and rehabilitation care to reduce avoidable hospitalisations and complications resulting from delayed diagnosis [48]. In sum, the registry serves as a management tool, guiding activities ranging from the design of expanded newborn screening programmes to the definition of clinical protocols and therapeutic guidelines for RDs within SUS.

In the realm of public financing, greater efficiency and transparency are expected. A national registry can be integrated with authorisation systems for high-cost procedures and medicines, accelerating verification of patient eligibility and monitoring of clinical outcomes (pharmacovigilance and effectiveness tracking). This enables policymakers to evaluate the return on investment of RD treatments: by linking medication-use data with registry-reported outcomes (e.g., improvements in quality of life or reductions in hospitalisations), they can negotiate risk-sharing models with manufacturers or reallocate funds toward interventions with greater demonstrated impact. Additionally, the transparency afforded by publicly available aggregated data (while safeguarding individual privacy) supports social accountability and more evidence-based advocacy by patient organisations, strengthening participatory governance within the health system.

### International Comparison and Applicable Lessons

Building on these expected impacts, it is helpful to examine how other countries have organised their RD registries and to draw lessons for Brazil. Several countries have already implemented consolidated RD registry systems, whose experiences offer valuable insights for Brazil.

A pioneer in RD policy since 2004, France developed Orphanet and implemented the National Rare Disease Database (BNDMR). The BNDMR collects standardised information (approximately 50 minimum data elements per patient) from all RD reference centres nationwide [52], enabling national visibility into the number of cases, average diagnostic delay, treatments used, and outcomes. This standardisation, supported by patient consent and authorisation from the French data protection authority (Commission nationale de l’informatique et des libertés, CNIL), facilitates both research and planning: France can periodically assess the impact of its National Rare Disease Plans using these data [52]. The key lesson for Brazil is the importance of defining a unified minimum data set and integrating specialised services into a shared information network, supported by robust legal and ethical frameworks [60].

Complementing the French national-registry model, the ERNs add an important network-based lesson for Brazil by connecting centres of expertise for rare, complex, and low-prevalence diseases across countries [64]. ERNs show that rare-disease registries are most useful when embedded in a coordinated ecosystem of expert centres, referral pathways, registry governance, and secure cross-institutional data use. Their alignment with the EU Rare Disease Platform and the European Rare Disease Registry Infrastructure illustrates how minimum data elements can support interoperable rare-disease registration [59]. The use of ORPHAcodes further reinforces semantic comparability across registries and health systems [50]. For Brazil, this model indicates that a national registry should not be designed merely as a stand-alone central database, but as part of an interoperable clinical and data-governance infrastructure.

Historically characterised by province or disease-specific registries, Canada has more recently launched a national strategy that includes establishing registries and developing inventories and standards. In 2024, the Canadian drug agency mapped 148 RD registries across the country, revealing heterogeneous formats and interoperability gaps [56]. Building on this, the Canadian Orphan Drug Strategy (2023) allocated funding to improve data collection and to develop tools to harmonise RD registries [55]. For Brazil, the Canadian experience underscores the need to first understand the landscape of existing registries, often dispersed across hospitals, universities, and patient groups, and then integrate these efforts under a unified structure to avoid duplication.

Beyond France and Canada, Spain and Italy offer mature examples of national rare disease registry infrastructures that are highly relevant to Brazil. In Spain, the development of the Spanish Rare Diseases Registries Research Network (SpainRDR) combined patient outcome registries with population-based registries, covering over 80% of the population in a pilot phase and identifying more than 800,000 rare disease cases [65]. Building on this, the Spanish Registry of Rare Diseases (Registro Estatal de Enfermedades Raras, ReeR) was formally implemented in 2015 as one of the first nationwide surveillance systems for chronic rare conditions, aggregating data from regional registries to support epidemiological monitoring and health-services planning [66, 67].

Italy followed a similar path by establishing the Registro Nazionale Malattie Rare (RNMR) in 2001 as a network of regional registries coordinated by the National Centre for Rare Diseases at the Istituto Superiore di Sanità, achieving full national coverage by 2011 [68]. Subsequent analyses have shown that the RNMR can be effectively linked to hospital discharge data and used to monitor the burden and outcomes of dozens of rare diseases [69, 70], while the new National Plan for Rare Diseases 2023–2026 further institutionalises the use of registry data for service planning and access to care [71].

In 2020, Australia implemented its National Strategic Action Plan for Rare Diseases, one of whose pillars is “data and registries.” A broad audit conducted in 2023 by Rare Voices Australia and Monash University found a lack of interoperability and coordination among Australian registries, while highlighting substantial benefits where they exist (e.g., improved outcomes and quality of care) [57, 58]. In response, recommendations for a national approach to RD data were published [57], proposing the creation of a central hub, adoption of minimum data sets, and sustainable funding. The central message from this experience is that even in high-income countries, strong central coordination is indispensable for isolated registries to reach their full potential. The Australian initiative also underscores the importance of engaging multiple stakeholders (government, researchers, industry, and patient organisations) in the governance of a future national registry.

In summary, international experiences converge on several guiding principles: (a) the need for strong governmental leadership and national plans that sustain registries over time (avoiding political and administrative discontinuities); (b) active patient involvement and person-centreed data use, ensuring practical utility at the point of care; (c) adoption of international data standards to enable global comparisons and access to innovations; and (d) financial and legal sustainability to ensure long-term viability of registries.

Applying these lessons to the Brazilian context will increase the likelihood of success and impact in mapping and integrating RD registries, ultimately resulting in tangible benefits for the millions of Brazilians affected and for the health system as a whole.

### Data Governance, Ownership, and Privacy Safeguards

A unified Brazilian rare-disease registry should not frame patient data as unrestricted private, institutional, or state property. In rare-disease registries, data ownership is better understood as a governance question involving stewardship, accountability, legitimate control, transparency, and participation in decisions about access and reuse. Patients and patient organisations should therefore be represented in governance boards, data-access committees, prioritisation of secondary uses, transparency policies, and mechanisms for returning aggregate findings to the community [24, 72].

This issue is particularly important because rare-disease data are difficult to anonymise fully. Low prevalence, granular phenotypes, genetic variants, geographic information, family history, and combinations of indirect identifiers may increase the risk of re-identification. Accordingly, record-level, longitudinal, linkable, clinical, genomic, or geographic registry data should generally be managed as sensitive personal data and, when linkage is needed, processed under pseudonymisation, role-based access control, encryption, audit trails, data minimisation, and re-identification risk assessment. Aggregated or effectively anonymised outputs remain appropriate for public reporting, epidemiological dashboards, and international dissemination when re-identification risk has been appropriately mitigated [13, 73, 74].

Brazil’s LGPD provides the national legal framework for processing sensitive health data, while the GDPR offers an important international comparator for concepts such as pseudonymisation, anonymous data, accountability, and privacy by design. These frameworks converge on the need for clear legal bases, proportionality, transparency, purpose limitation, security safeguards, and governance mechanisms for secondary use of health data [13, 73]. The Global Patient co-Owned Cloud (GPOC) literature is relevant in this context because it conceptualises patient-centred control, co-ownership, and co-stewardship of personal health information in a fragmented global data environment. Although a GPOC-style personal health record model cannot be transferred uncritically to the Brazilian SUS context, it provides a useful conceptual reference for designing a federated registry governance model that combines public-interest stewardship with patient participation and controlled data reuse [75].

### Study Recommendations

To overcome fragmentation among national, state-level, and condition-specific initiatives, the combined adoption of four standardisation pillars is recommended: (i) the International Classification of Diseases, 11th Revision (ICD-11) for morbidity and mortality coding; (ii) ORPHAcodes for granular identification of RDs; (iii) the Common Data Elements (CDEs) proposed by the European Platform on Rare Disease Registration; and (iv) data exchange through Health Level Seven (HL7) Fast Healthcare Interoperability Resources (FHIR) profiles (R5 or later) to ensure technical interoperability. ICD-11 entered into force globally on 1 January 2022, and the WHO released an update in 2025. In Brazil, implementation began in 2021 and is expected to conclude by 2027 [51, 76, 77].

The use of ORPHAcodes, an internationally recognised terminology for RDs helps address under-representation in clinical coding and enhances interoperability across databases [49, 50]. For minimum content, the European CDEs define 16 essential elements (such as patient identification, vital status, diagnosis, disease history, and care pathway), forming a core dataset that can be readily harmonised with the Brazilian context [59, 78].

From a technical perspective, it is recommended that national FHIR profiles be published (e.g., Condition, Observation, Patient, Encounter, and List), aligned with ICD-11 and ORPHAcodes, along with an Implementation Guide defining terminologies, cardinalities, and versioning mechanisms [79, 80]. This enables integration with electronic health records, laboratories, genomics initiatives (Genomas Brasil), and administrative systems such as the Hospital Information System of SUS (SIH/SUS) and the National Registry of Health Establishments (CNES), while preserving governance mechanisms and audit trails.

From the governance standpoint, LGPD requirements and the legal bases for data processing must be observed in the unification process, including clear designation of the controller and processor, appointment of a Data Protection Officer (DPO), evaluation of legal bases (execution of public policies and protection of health), mapping of sensitive data, and development of a Data Protection Impact Assessment (RIPD) specific to RDs [13, 14]. The preferential legal basis for processing within SUS is the execution of public policies, which may be complemented by the health protection basis and by legitimate interest in specific contexts (with safeguards). Informed consent remains recommended when data are collected directly for non-mandatory purposes, to ensure transparency and the option to opt out of secondary uses.

The proposed architecture should integrate with the National Programme for Genomics and Precision Health, Genomas Brasil, whose regulation was updated in 2025, expanding scientific community participation and infrastructure for precision public health in SUS [30, 31, 81]. Technical-semantic integration may be achieved by linking laboratory results and genetic variants to ICD-11/ORPHA-coded diagnoses via specific FHIR profiles (e.g., MolecularSequence/Observation- genetics), while preserving security layers and differential access governance for sensitive genomic data.

The European experience with the EU RD Platform demonstrates that defining CDEs and adopting standardised terminologies reduces fragmentation and enables multicentre research [59, 82]. In parallel, WHO recommends the rapid implementation of ICD-11, which has already been adopted or is in transition in several countries, reinforcing international convergence in RD coding [51]. For Brazil, the following are recommended: (i) a national CDE guide adapted to SUS realities; (ii) mandatory use of ORPHAcodes in accredited services;

(iii) federal incentives for technical (FHIR) and semantic (ICD-11/ORPHA) integration; and (iv) cooperation with international networks for secure data and methodological sharing.

A national data-quality programme is proposed, including periodic audits (completeness, consistency, and timeliness), feedback to centres, and workflow adjustments to reduce underreporting. Compliance metrics (e.g., the percentage of mandatory fields completed and the rate of ICD-11/ORPHA coding) should serve as public transparency indicators and drive continuous improvement.

### Study Limitations

This descriptive mapping study is subject to underreporting of regional or institutional initiatives not documented in public sources, as well as omissions arising from the terminological diversity used to name systems and databases. Because the design was not a conventional systematic review, the results should be interpreted as an auditable mapping of registry entities and registry-related initiatives rather than as an exhaustive synthesis of intervention, diagnostic, prognostic, or treatment-effectiveness evidence. The focus on official documents and scientific publications may fail to capture ongoing or informal projects. Future research may combine direct surveys with service and manager stakeholders, stakeholder interviews, and technical interoperability audits to improve the comprehensiveness of mapping and strengthen data-quality assessment.

## Conclusion

The panorama presented in this study indicates that Brazil already has a substantive foundation for unifying RD registry entities and registry-related initiatives. Still, it lacks primarily political coordination, technical and informational standardisation, and integration among existing systems. The proposed unification must articulate, within an interoperable architecture, the primary data sources currently dispersed: epidemiological evidence from the RARAS Network and the Raras Brasil Project [5], state-level notification systems (e.g., SIDORA), hospital and specialised service records, information on congenital anomalies from SINASC, CONITEC’s technology-incorporation decisions, and identification databases associated with state health cards. Consolidation of these layers under international terminologies (ICD-10/ICD-11 and Orphanet) and interoperability standards will enable the reconstruction of an accurate national overview of the prevalence, incidence, and geodemographic distribution of rare conditions, thereby improving policy planning and evaluation.

In the governance domain, a centralised arrangement linked to the Ministry of Health is recommended, with formal participation from universities of the RARAS Network, state health secretariats, and reference services, thereby ensuring that the Observatory of Rare Diseases at the University of Brasília (UnB) assumes a coordinating technical-scientific role. A clear legal basis should underpin this design, particularly the approval and operationalisation of Bill 109/2025, which establishes the SNMDR, as well as safeguards for security, privacy, pseudonymisation of record-level data, and aggregation or effective anonymisation for public reporting in compliance with the LGPD. Implementation should be gradual and cumulative, progressively integrating existing systems and legacy datasets, with continuous training programmes for registry teams and social-engagement strategies to reduce underreporting.

A unified national registry would therefore be more than an administrative instrument: it would provide the data infrastructure needed to reduce fragmentation, support equitable access to care and benefits, strengthen research capacity, and improve policy monitoring. If implemented gradually, with technical rigour, social participation, and robust legal safeguards, this integration could position Brazil as a reference in Latin America for rare-disease information governance and care.

## Supplementary Information

Below is the link to the electronic supplementary material.

Supplementary Material 1 (XLSX 11.8 KB)

Supplementary Material 2 (XLSX 6.21 KB)

Supplementary Material 3 (DOCX 326 KB)

Supplementary Material 4 (XLSX 18.1 KB)

Supplementary Material 5 (XLSX 18.1 KB)

## Data Availability

The data underlying this mapping study are available as supplementary material. Supplementary Table S1 contains the extracted inventory of the 28 rare-disease registry entities and registry-related initiatives identified in Brazil, including territorial scope, implementation status, responsible organisation, category, source document, URL or citation, access date, and rationale for inclusion. Supplementary Table S2 provides the source-specific search strategies and included-source auditability log and screening documentation. Dataset S1 contains the extracted mapping spreadsheet in machine-readable format. Dataset S2 contains the included-source auditability log. No individual-level patient data were analysed or shared; all extracted information was derived from public-domain documents, official sources, registry portals, legislative documents, institutional reports, or scientific publications.
