# Supplementary figures and images for: Mapping Rare Disease Registries in Brazil: Situational Analysis and Proposal for National Unification
Source: J Med Syst. 2026 Jul 20;50(1):115. doi: 10.1007/s10916-026-02442-w (PMC13385086; doi:10.1007/s10916-026-02442-w)

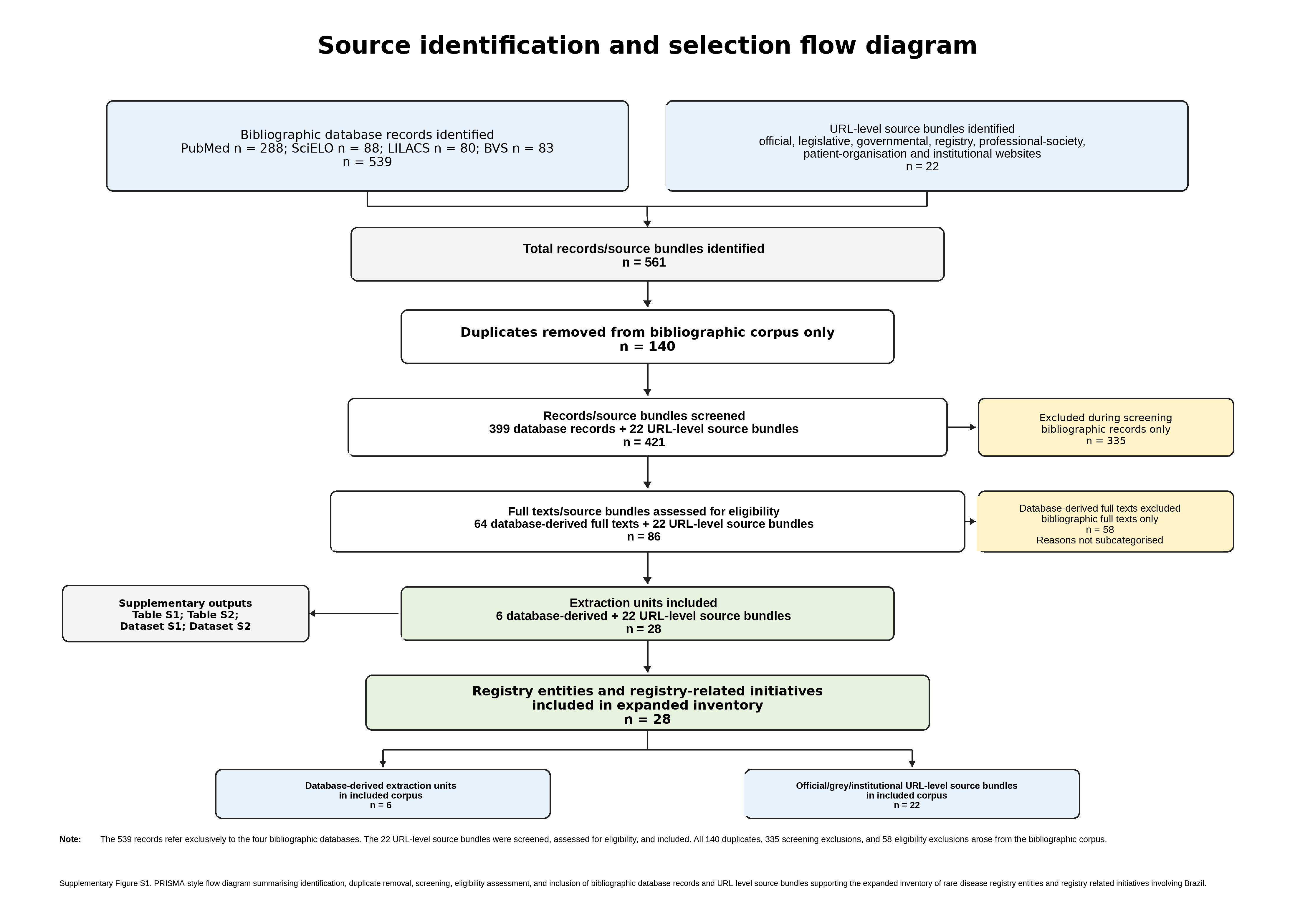

Supplement: Supplementary file 3 — Supplementary Material 3 (DOCX 326 KB) [file 10916_2026_2442_MOESM3_ESM.docx]
